# Abrasivity behavior analysis and fuzzy stochastic prediction of weakly cemented sandstones using an improved RBF neural network for quantifying uncertainties

**DOI:** 10.1371/journal.pone.0345942

**Published:** 2026-04-07

**Authors:** Yafeng Yao, Jian Lin, Xiangwei Li, Yongheng Li

**Affiliations:** 1 School of Architecture and Engineering, Nantong Vocational University, Nantong, China; 2 Department of Civil Engineering, Anhui Jianzhu University, Hefei, China; 3 College of Civil Engineering, Huaqiao University, Xiamen, China; Henan Polytechnic University, CHINA

## Abstract

Uncertainties in rock abrasivity often result in the failure of mechanical excavation and excessive cutter wear during TBM tunnelling. Cerchar abrasivity characteristic tests on weakly cemented sandstones (WCS) in western China revealed that their abrasivity changed with physical state and exhibited fuzzy randomness. In the dry state, the abrasivity index reaches its maximum, with the mud-saturated state being intermediate, and the water-saturated state showing the lowest value. In the dry state, the sandstone’s Cerchar Abrasivity Index(CAI) increased (CAI) by 10.64% compared to the mud-saturated state, and by 19.23% compared to the water-saturated state. Studies on the microstructure of WCS in different states indicated that the higher CAI value in the dry state is attributable to its well-preserved internal structure, strong cementation, and high strength. In the water-saturated state, the presence of a slurry film on the sandstone surface led to a higher steel stylus wear than in the fully saturated state. The conventional RBF neural network model was improved by introducing neurone fuzzy splitting and stochastic adjustment of connection weights. Compared to MLR, SVR, and traditional RBF: the MSE of improved RBF is reduced by up to 65.7%, the MAE is reduced by up to 47.4%, and the occurrence rate of local optimality is decreased by 53.8%. Based on this, an improved fuzzy stochastic RBF neural network model was established to predict rock abrasivity using hardness, wave velocity, porosity, and equivalent quartz content as inputs, and the CAI and Cerchar Abrasivity Ratio (CAR) of WCS as outputs. Engineering examples show that the improved RBF fuzzy stochastic model’s prediction of rock abrasivity rate has an error of less than 5% compared to the actual measured values, and the predicted Coefficient of Determination (R^2^) reaches 0.967. Therefore, the enhanced prediction model successfully addressed the uncertainty in abrasivity characteristics of WCS in western China.

## 1. Introduction

Since the beginning of the 21st century, with the implementation of major national projects such as the Western Development Strategy, the initiative to build a strong transportation network, and the South-to-North Water Diversion Project, tunnel construction has become a crucial component of infrastructure development [1–2]. Currently, the innovative rock-breaking technologies adopted in the tunnelling sector primarily consist of the drilling rig technique and the Tunnel Boring Machine (TBM) full-face excavation method [3–6]. However, at the current stage, due to the TBM’s poor adaptability to varying rock and geological states, severe uncertainty arises in cutter wear when encountering heterogeneous strata, highly abrasive hard rocks, and complex formations. Consequently, cutterhead replacement cannot be predicted accurately, leading to low TBM excavation efficiency, construction delays, and significant increases in project costs [7–14]. It is evident that the abrasiveness of rock not only reflects its fundamental physical and mechanical properties but also serves as a critical prerequisite for accurately predicting the TBM rock-breaking efficiency and disc cutter wear. Currently, the Cerchar abrasivity test is an internationally accepted method for evaluating rock abrasiveness. It determines rock abrasiveness based on the wear of a steel stylus and classifies it according to the Cerchar Abrasivity Index (CAI) [15]. Numerous experimental studies have shown that CAI is closely related to the compressive and tensile strengths of rock [16–18]. Moreover, the mineralogical composition and microstructural characteristics of the rock affect its CAI value [19–21].

Numerous studies and discussions on rock abrasivity have been conducted by scholars both domestically and internationally: Mehmet Capik [22–23] established the relationships between the CAI and uniaxial compressive strength, Schmidt rebound hardness, and point load strength using simple and multiple linear regression analyses. Plinninger et al. [24] discovered that the wear values measured using the Cerchar scratch test cannot be explained solely by the equivalent quartz content. Beste et al. [25–26] demonstrated that the size of quartz grains plays a critical role in scratch wear, with smaller grains causing lower wear rates and larger grains resulting in higher wear rates. Ahmet Teymen [27–28] investigated 80 different types of rocks to evaluate the Young’s modulus, uniaxial compressive strength, and CAI to estimate key mechanical rock properties that are otherwise difficult and time-intensive to determine using CAI as an indicator. He further established multivariate regression Equations linking CAI to several parameters. Kahraman et al. [29] used regression analysis and artificial neural networks (ANNs) to analyse data related to CAI and developed indirect models for predicting CAI based on uniaxial compressive strength (UCS) and elastic modulus (E). Tae Young Ko et al. [30] investigated the relationship between CAI and geomechanical properties using both univariate and multivariate regression analyses and observed that individual parameters are not suitable for predicting CAI and developed a CAI prediction model based on geomechanical characteristics.

In summary, previous studies on rock abrasivity mostly relied on experimental data analysis and regression formulas without considering the pronounced uncertainty in the abrasivity distribution under the combined effects of complex underground geotechnical states, leading to deviations in the results [31–35]. Moreover, although artificial neural networks have been used to analyse CAI-related data, traditional intelligent algorithms cannot adequately address fuzzy stochastic ness in engineering applications, necessitating targeted algorithm improvements [36–39].

## 2. Sample preparation and selection of WCS

### 2.1 Sample preparation

**Fig 1:**
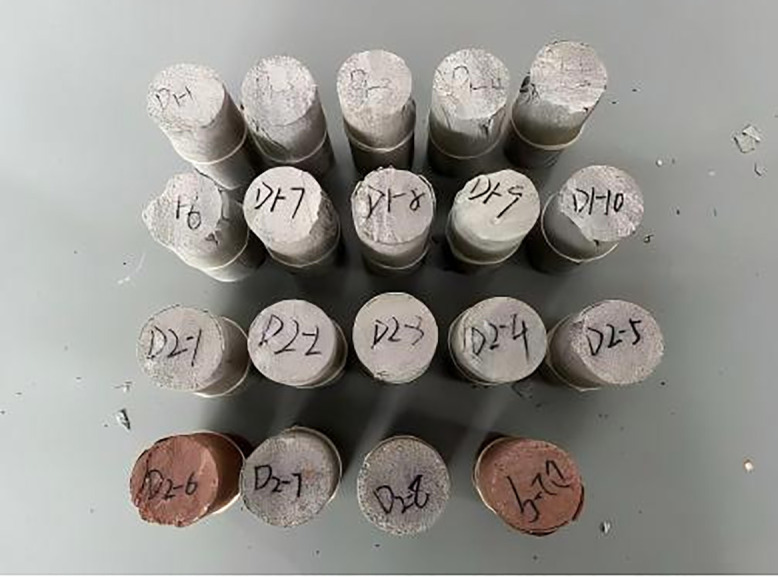
Core sample.

**Fig 2:**
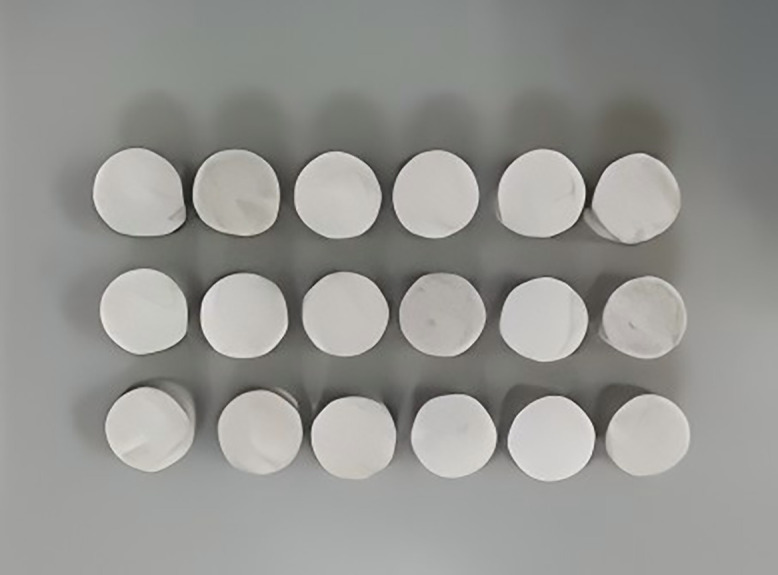
Test samples.

The weakly cemented rock samples used in this study were obtained from the TBM tunnel project of the Balasu Coal Mine in Yulin City, Shaanxi Province. According to a geological report, the strata are composed primarily of mudstone, sandy mudstone, and sandstone, which are characteristics of typical Jurassic formations. The rock samples collected from the site were sealed with plastic wrap and transported to the laboratory, where they were processed, as shown in Fig 1. According to the method recommended by the International Society for Rock Mechanics and Rock Engineering (ISRM), and in combination with the structure and clamping requirements of the testing instruments used in this study, all the samples were processed into standard cylindrical shapes of φ50 mm × 25 mm. To prevent eccentric loading during the test from affecting the experimental data, the ends of the specimens were ground after processing to ensure that the flatness error was controlled within ±0.2 mm. Prior to the testing, no additional materials were used to wrap the ends of the sandstone specimens to avoid affecting the flatness of the end surfaces. The prepared specimens are shown in Fig 2.

### 2.2 Selection of WCS samples

Rock density, defined as the mass per unit volume, including pore space, is a fundamental physical parameter that reflects the mineral composition and content, porosity, number of pore fillings, and stress level within the rock. Conventional physical parameter measurements were conducted on a previous batch of rock samples. An electronic balance and Vernier caliper were used to measure the mass and volume of the WCS in its natural state, and to calculate its natural density.

**Fig 3:**
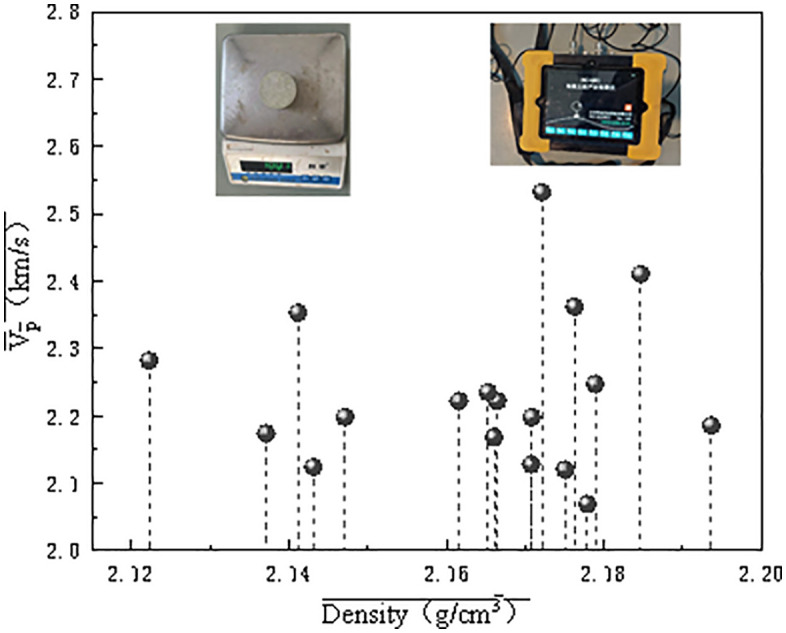
Density–Velocity of WCS Samples.

After conducting the density tests on the processed rock samples, wave velocity tests were performed on the sandstone to ensure that the test specimens had no end-face irregularities or cracks. The velocity of longitudinal waves in a rock, which is a fundamental physical parameter, indicates the degree of jointing and fracturing within the rock and the overall rock mass integrity. In longitudinal waves, the particle motion is parallel to the direction of wave propagation. According to the GB 50021−2001 *Code for Investigation of Geotechnical Engineering*, the primary method for measuring rock-mass wave velocities is ultrasonic testing. An HC-U81 ultrasonic device was used to measure the longitudinal wave velocities of the selected samples. The rocks are numbered based on the results. The density–velocity screening results are shown in Fig 3.

## 3. Cerchar abrasivity test

The abrasivity of rocks directly reflects the difficulty and efficiency of rock-breaking. This serves as an important reference for evaluating the wear of cutter rings and tools in drilling and TBM construction. Generally, the higher the rock abrasivity, the greater the wear on the tools and cutter rings, leading to increased tool consumption and higher construction costs. Factors affecting rock abrasivity include mineral content, hardness, rock texture, particle size and shape, interparticle bonding, and fundamental physical and mechanical properties.

**Fig 4:**
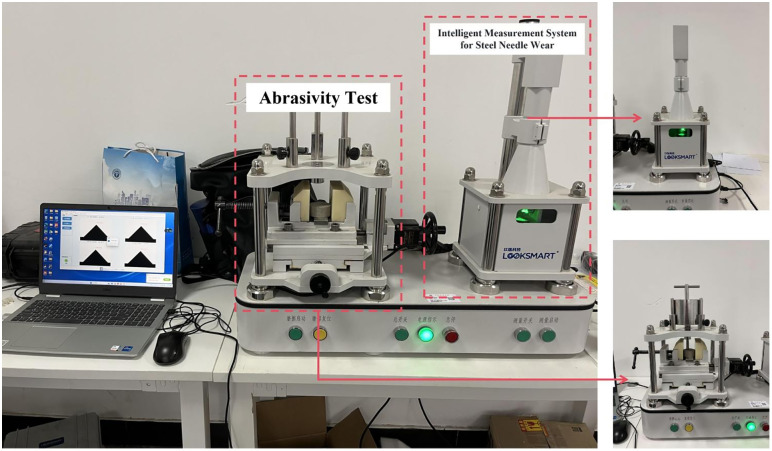
Cerchar rock abrasivity test apparatus.

The cerchar rock abrasivity testing apparatus was used in this rock abrasivity test, which consisted of two main components: a rock friction tester and an intelligent steel needle wear measurement system. In this test, the rock sample was fixed on a sample holder, and a steel needle, with a tensile strength of 2 GPa, Rockwell hardness (HRC) of 54–56, and a tip angle of 90° was placed above the sample. A force of 70 N was applied to the needle and a stepper motor drove the module to move the needle 10 mm across the sample surface within one minute. The wear at the needle tip was measured using an intelligent wear measurement system and converted into the CAI. The friction tester included a sample holder, two handles, a stepper motor, a ruler, a steel needle, a needle fixture, and a fixed load (7 kg). The intelligent wear measurement system consisted of a machine vision measurement system and a needle-holding device. The testing apparatus is shown in Fig 4.

Before testing, the pre-numbered rock samples were processed in three states: dry, water-saturated, and mud-saturated. The equipment was inspected at the shaft and threaded sections and lightly lubricated with machine oil. The quality of the steel needle tip was verified, and the needles met the test requirements.

**Fig 5:**
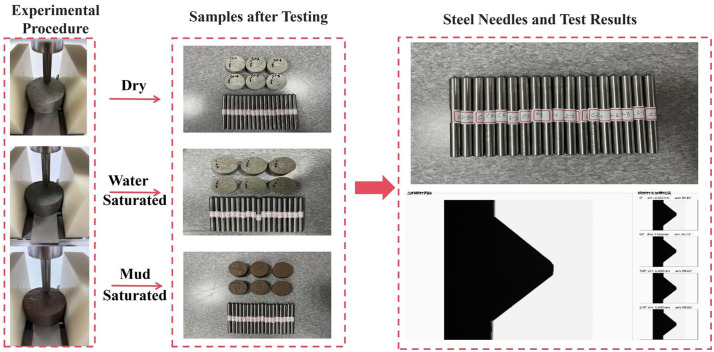
Schematic diagram of the abrasivity test procedure.

A steel needle was gently placed in the upper groove of the sample, and a fixed load of 70 N was applied above the needle. The friction start button was pressed to control the stepper motor, causing the needle to move uniformly at a speed of 10 mm/min on the sample surface. After testing, the load was removed and the needle was removed and labelled. Each sample was subjected to three scratch tests. We input the relevant information of the rock sample and steel needle into the software interface, removed the debris from the needle tip after testing, and inserted it into the intelligent measurement device. Upon initiating the measurement, the system performed four measurements by rotating the needle 90° between each test and recorded the diameters of the four worn surfaces (with a precision of 0.001 mm), denoted as d₁, d₂, d₃, and d₄. Fig 5 shows the worn tip diameters along with the tested sample and steel needle.

Based on the specimen preparation steps described above, the CAI for the WCS in various states was calculated using the measured steel needle wear and Equation (1):


CAI=10×d (1)

**Fig 6:**
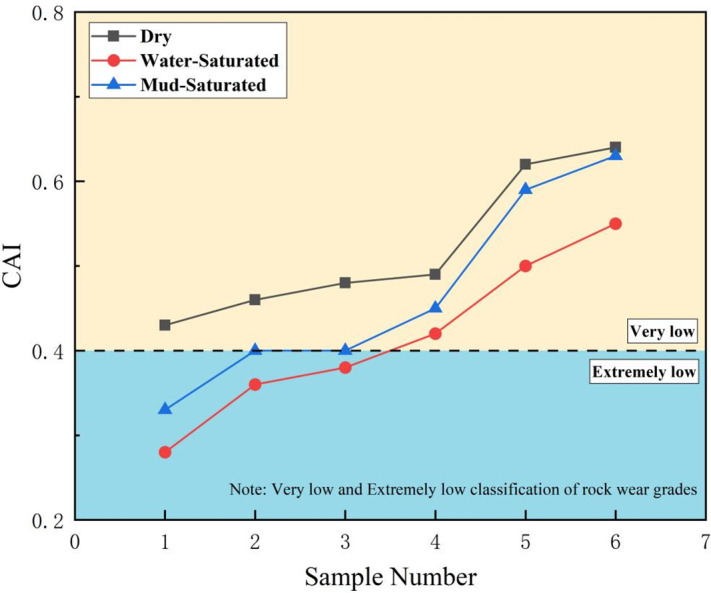
Summary of sandstone CAI values of different states.

**Fig 7:**
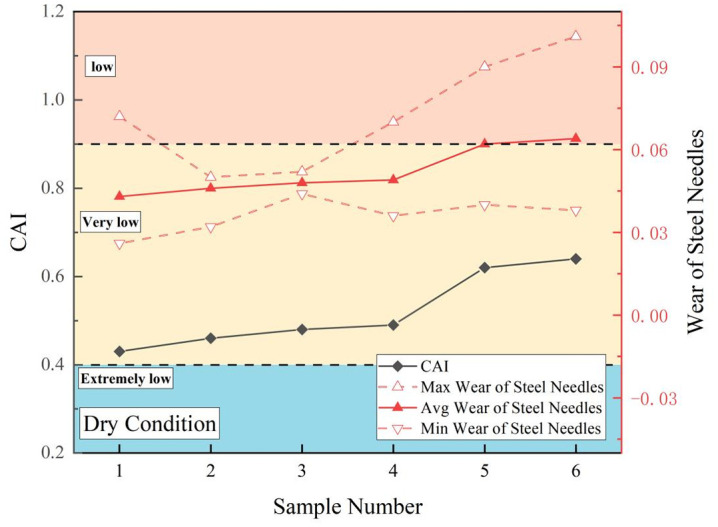
CAI values and steel needle wear of sandstone under dry states.

**Fig 8:**
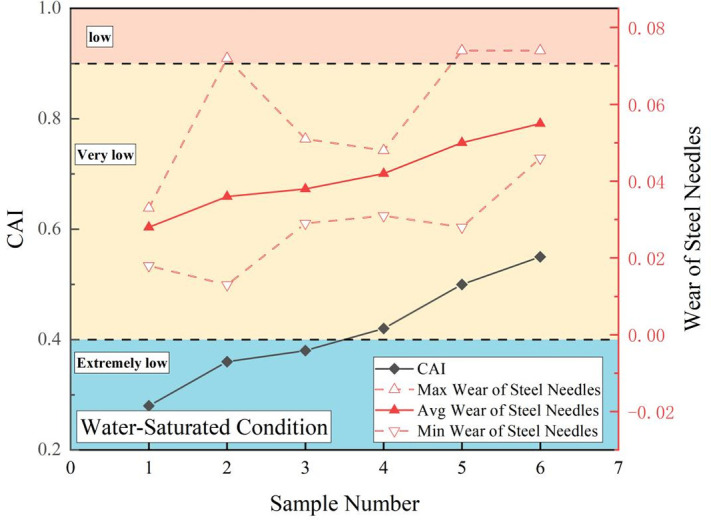
CAI values and steel needle wear of sandstone under saturated states.

**Fig 9:**
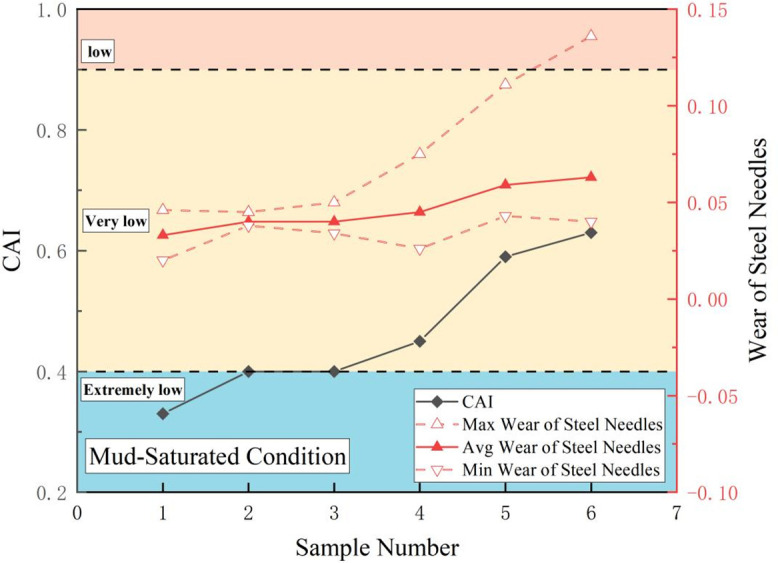
CAI values and steel needle wear of sandstone under mud-saturated states.

In Equation (1), *d* denotes the abrasivity width (mm) of the steel needle.The coefficient 10 is a conversion factor specified by the standard test method (to convert the abrasivity width in mm to a dimensionless index with clear grading significance). Figs 6–9 show the maximum, minimum, and average CAI values (dimensionless) and wear measurements for sandstone in each state. The CAI in the dry state exceeded that in both the water-saturated and mud-saturated states.

We performed a statistical analysis of the CAI values of the WCS in different states, deriving the maximum, minimum, and mean values. The abrasivity of the sandstone in each state was assessed according to the ISRM CAI classification criteria, as listed in Table 1. As listed in Table 1, the abrasivity of WCS varies with its state. Overall, it was highest in the dry state, followed by the mud-saturated state, and lowest in the water-saturated state, with evident uncertainty. The CAI value of sandstone reached a maximum of 0.64 and a minimum of 0.43 in the dry state, a maximum of 0.55 and a minimum of 0.28 in the water-saturated state, and a maximum of 0.63 and a minimum of 0.33 in the mud-saturated state. On average, the CAI value was lowest for saturated sandstone at 0.42 and highest in the dry state at 0.52, indicating a 19.23% increase over the water-saturated state. The mud-saturated sandstone had an intermediate average CAI of 0.47, which was 10.64% higher than that of the water-saturated state and 10.64% lower than that of the dry state.

**Table 1:** Statistical and evaluation results of sandstone CAI values under different states.

State	Dry	Water-saturated	Mud-saturated
Maximum value	0.64	0.55	0.63
Minimum value	0.43	0.28	0.33
Average value	0.52	0.42	0.47
Abrasivity level	Very low	Very low	Very low

Notes:The CAI grading follows the ISRM suggested methodfor rock abrasivity evaluation. This standard is widely recognized in rock engineering and TBM tool abrasivity prediction.

## 4. Analysis of abrasive efficiency of WCS under different states

The CAI reflects the mechanical interaction between the steel needle and rock. Under a constant load of 70N, the steel needle scratched across the rock surface and underwent wear. In engineering practice, CAI testing is critical for evaluating the tool wear and economic performance. To further investigate the abrasivity characteristics of the steel needle in WCS, 3D surface scanning can be employed to obtain the wear volume during tests under various states, and the interaction between sandstone and the needle can be evaluated via the Cerchar Abrasivity Ratio (CAR), which is defined as the ratio of the needle wear volume to the volume of removed sandstone.

### 4.1 Measurement of abraded sandstone volume

**Fig 10:**
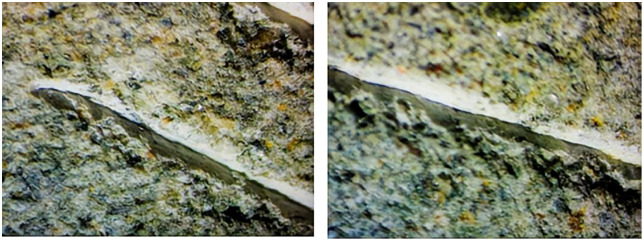
Wear surface of sandstone under dry state.

**Fig 11:**
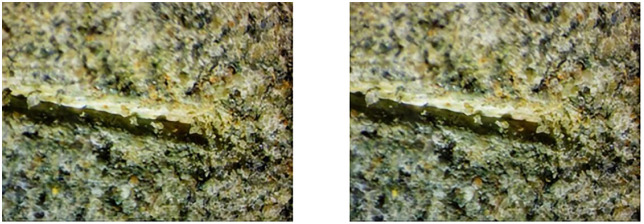
Wear surface of sandstone under water-saturated state.

**Fig 12:**
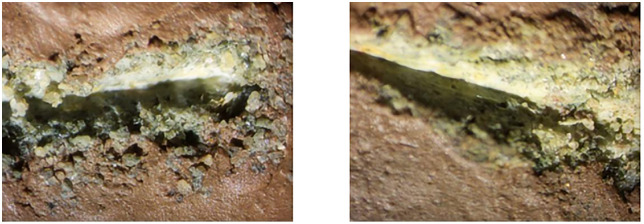
Wear surface of sandstone in mud-saturated state.

A microscope was employed to closely examine the scratch grooves formed on the sandstone surface after the Cerchar abrasivity test to better understand the surface damage induced by the steel needle. Figs 10–12 shows the scratch groove damage on the sandstone surface after the abrasivity test. As shown in Figs 10–12, the steel needle penetrates the sandstone surface under a fixed load of 70 N and forms a distinct V-shaped groove under horizontal thrust. The presence of worn debris particles within the groove indicated that the rock grains were dislodged by the steel needle. Weak bonding between grains was primarily responsible for the detachment of adjacent large particles during the needle abrasivity process. Consequently, the scratched grooves on the surfaces were uneven.

**Fig 13:**
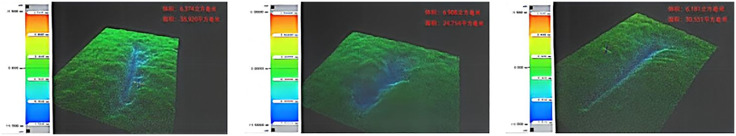
3D surface morphology scan results of sandstone in dry state.

**Fig 14:**
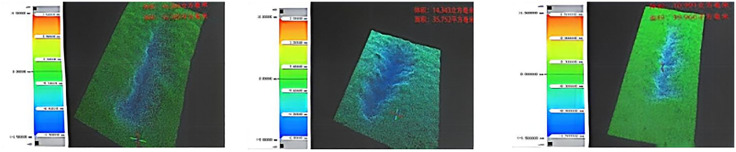
3D surface morphology scan results of sandstone in water-saturated state.

**Fig 15:**
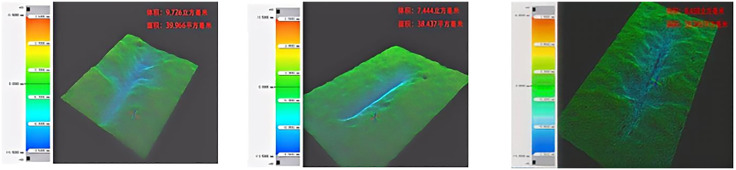
3D surface morphology scan results of sandstone in mud-saturated state.

To further investigate the abraded-surface material volume of sandstone, 3D profilometry was conducted on the grooves formed under three different states. Because of the structural similarity and large number of scratches, it was impractical to scan them all; therefore, three representative scratches from each state were selected for 3D scanning and observation. The detailed results are shown in Figs 13–15. As shown in Figs 13–15, the abraded volume of sandstone under dry states ranged from 6.181 mm³ to 6.908 mm³, with an average of 6.488 mm³; under water-saturated states, it ranged from 10.991 mm³ to 16.266 mm³, averaging 13.867 mm³; and under mud-saturated states, it ranged from 7.444 mm³ to 9.726 mm³, with an average of 8.453 mm³. The average abraded volume generally followed the trend: water-saturated state > mud-saturated state > dry state. However, the data exhibit a degree of irregularity and stochastic dispersion. Compared to the low-strength, low-abrasivity water-saturated state, the average abraded volume of sandstone decreased by 38.39% and 53.21% in the high-strength, high-abrasivity dry state. Compared to the mud-saturated state, the dry state exhibited a 24.06% reduction in average abraded volume. This is because the water-saturation reduces the apparent hardness of sandstone. Under a fixed load applied to the steel needle, this leads to a greater penetration depth and increased contact area while simultaneously lowering the compressive and tensile strengths of the sandstone. Consequently, the abraded volume of sandstone in the water- and mud-saturated states exceeded that of the dry state. Furthermore, the mud has a viscosity that is absent in water and coats the sandstone surface during saturation. Therefore, the steel needle must first traverse the mud layer before penetrating the rock. As it advances under a horizontal force, part of the resistance encountered by the needle originates from the viscous mud layer. This explains why the average abraded volume in the mud-saturated state is lower than that in the water-saturated state.

### 4.2 Calculation of the CAR

The abrasivity volume of the sandstone varied under three different states. To better characterise the abrasivity efficiency of sandstone, a new abrasivity index called the CAR (dimensionless) is introduced, which relates to two volume parameters. The formula is as follows:


$$CAR=\frac{V_{m}}{V_{s}}$$ (2)

In the formula:

$V_{m}$ — abrasivity volume of sandstone, mm^3^;

$V_{s}$ — abrasivity volume of steel needle, mm^3^.

The 3D surface scans described above provide the abraded volume of sandstone. For the steel needle tip (to determine *V*_*s*_), 3D surface characterization was performed using a confocal laser scanning profilometer (Olympus LEXT OLS5000). A 0.5 mm × 0.5 mm area (covering the conical abrasivity region) was scanned with a lateral resolution of 0.05 μm/pixel, vertical resolution of 0.01 μm, and step size of 0.1 μm. Raw data were filtered with a Gaussian filter (cut-off: 0.2 mm) to remove noise. *V*_*s*_ was calculated by: (1) scanning the unworn needle tip as a conical reference; (2) aligning the worn and reference scans; (3) extracting the height reduction h (maximum vertical difference between worn/reference surfaces); (4) fitting the abrasivity region to a truncated cone (Equation (3)) using OLS5000 Analysis software. The diameter d of the needle tip was confirmed via the unworn reference scan. The abrasivity volume *Vs* is expressed as follows:


$$V_{\text{s}}=\frac{\text{1}}{\text{3}}\pi (\frac{d}{\text{2}}{)}^{\text{2}}h$$ (3)

In the formula:

*h* — the height reduction of the steel needle tip after the abrasivity test, mm.

Because the Cerchar abrasivity Ratio (CAR) is related to both the steel needle tip wear volume and the abraded sandstone removal volume, it can be used to describe the interaction between the sandstone and steel needle. As listed in Table 2, for the high-strength and highly abrasive sandstone in the dry state, the Cerchar abrasivity Ratio (CAR) reached its minimum among the three tested states. This is attributed to the reduced sandstone abraded volume (V_m_) and increased steel needle wear volume (V_s_). In the case of mud-saturated sandstone, both the strength and abrasiveness were reduced. Owing to the presence of the surface mud coating, Vm increased only slightly, while Vs decreased, resulting in a notable increase in the CAR compared to the dry state. For the saturated sandstone without mud coating, the decrease in strength and abrasiveness was accompanied by a significant increase in Vm and a substantial reduction in V_s_, leading to a markedly higher CAR than that in both the dry and mud-saturated states. Apart from the general stochastic distribution of the CAR, the study found that the abrasivity rate of the steel needle was lower when acting on saturated sandstone compared to the dry and mud-saturated states. Moreover, in the Cerchar abrasivity tests of WCS from the western regions, the abrasivity efficiency, represented by the CAR, was the highest in the water-saturated state (CAR = 4.259), followed by the mud-saturated state (CAR = 0.863), and lowest in the dry state (CAR = 0.405). These results provide a reference for evaluating the wear of mechanical rock-cutting tools.

**Table 2:** Abrasivity parameters of sandstone in different states.

Rock states	V_m_ (mm^3^)	V_s_ (mm^3^)	CAI	CAR	Average CAR
Dry	6.374	10.402 × 10^−6^	0.43	0.613	0.405
	6.181	15.392 × 10^−6^	0.49	0.402	
	6.908	34.307 × 10^−6^	0.64	0.201	
Water-saturated	10.991	2.872 × 10^−6^	0.28	2.592	4.259
	14.343	9.693 × 10^−6^	0.42	0.873	
	16.266	2.177 × 10^−6^	0.55	4.468	
Mud-saturated	7.444	4.702 × 10^−6^	0.33	2.338	0.863
	8.458	11.922 × 10^−6^	0.45	1.203	
	9.726	32.715 × 10^−6^	0.63	0.497	

**Notes**:The CAI grading follows the ISRM suggested methodfor rock abrasivity evaluation.The CAR represents the ratio of the abrasivity volume of sandstone to the volume of abrasivity volume of steel needle. A higher CAR value indicates that the rock causes more severe tool wear per unit volume of excavation, which is a key index for evaluating the rock’s abrasion efficiency.

In conclusion, in the face of complex and uncertain geotechnical environments, it is essential to employ artificial intelligence algorithms enhanced with fuzzy stochastic approaches to better understand rock abrasivity behaviour during TBM construction and improve the accuracy of abrasivity prediction in heterogeneous formations.

## 5. RBF fuzzy-stochastic neural network

### 5.1 Conventional RBF neural network architecture

In 1988, during the continuous development of neural network models, Lowe and Broomhead first proposed a Radial Basis Function (RBF) neural network by applying a multivariate interpolation method based on the RBFs introduced by Powell in 1985. The RBF neural network is a three-layer feedforward network characterised by excellent generalisation, high precision in approximating nonlinear functions, simple structure, and fast learning capability. It is well-suited for analysing and solving problems with uncertain input-output relationships.

**Fig 16:**
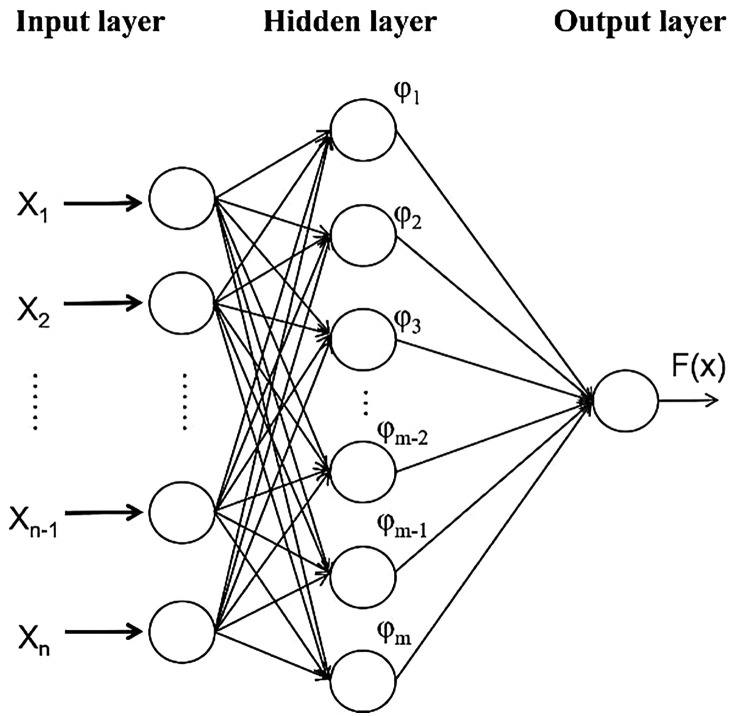
Topological Structure of the RBF Neural Network.

As shown in Fig 16, the RBF neural network consists of three layers: an input layer, a hidden layer, and an output layer. The relationship between the input and hidden layers is a nonlinear mapping, and the connection weights between them are set to one. The signal transmission between the hidden and output layers was linear and the final network output was obtained through a linearly weighted sum of the hidden layer outputs based on the connection weights. To enhance the weight estimation and learning speed while avoiding local minima, linear Equations or the recursive least squares method are commonly employed for training [40].

For the multivariate interpolation problem, given a set of N distinct points {*x*_*i*_ ∈ R_n_|*i* = 1,2,……,N} and their corresponding set of points *y*_*i*_ ∈ R_m_|*i* = 1,2,……,N}, Powell proposed the radial basis function formulation to satisfy the following states:


$$Y_{i}=F\left(x_{i}\right)$$ (4)

In an RBF neural network, all basis functions use the same radial basis function. Through N hidden layer neurones, all samples were input as the data centres of the radial basis functions. The network fits the input-output relationship by training to determine the output weights. When the input is x_i_, the output of the kth hidden layer node is expressed as follows:


$$h_{ik}=\varphi_{k}\left(\|x_{i}-\mu_{i}\|\right)$$ (5)

In the formula:

$\varphi_{k}$ is the radial basis function of the hidden layer node;

$\mu_{i}$ is the data centre of the RBF network.

The following are several commonly used radial basis functions:

(1) Standard Gaussian radial basis function:

$$g\left(x_{i}-\mu_{i}\right)=exp\left(-\frac{\text{1}}{{\sigma_{i}}^{\text{2}}}\|x_{i}-\mu_{i}\|\right)$$ (6)

(2) Inverse multiquadric radial basis function

$$g\left(x_{i}-\mu_{i}\right)=\frac{\text{1}}{1+exp\left(\frac{{\|x_{i}-\mu_{i}\|}^{\text{2}}}{{\sigma_{i}}^{\text{2}}}\right)}$$ (7)

(3) Cauchy radial basis function:

$$g\left({\text{x}}_{i}-\mu_{i}\right)=\frac{\text{1}}{1+\frac{{\|x_{i}-\mu_{i}\|}^{\text{2}}}{{\sigma_{i}}^{\text{2}}}}$$ (8)

(4) Inverse multiquadric function:

$$g\left(x_{i}-\mu_{i}\right)=\frac{\text{1}}{\sqrt{1+\frac{{\|x_{i}-\mu_{i}\|}^{\text{2}}}{{\sigma_{i}}^{\text{2}}}}}$$ (9)

Gaussian functions are commonly used as RBFs in the RBF networks. This study also adopted a Gaussian function as the radial basis function for the network model. The output of the RBF neural network structure model can be expressed as follows:


$$y=\sum_{n=1}^{\text{N}}\omega_{n}\varphi_{n}\left(x\right)$$ (10)

In the formula:

x represents the input value;

N denotes the total number of neurones in the hidden layer;

$\omega_{n}$ represents the weight of the nth hidden layer neurone;

$\varphi_{n}$ represents the radial basis function of the nth neurone in the hidden layer.

Based on the selection of the above radial basis functions, the final output expression of the RBF can be written as follows:


$$y=\sum_{n=1}^{N}\omega_{n}exp\left(-\frac{\text{1}}{{\sigma_{n}}^{\text{2}}}\|x_{n}-\mu_{n}\|\right)$$ (11)

In the formula:

$\sigma_{n}$ represents the variance of the nth hidden layer neurone;

$\mu_{n}$ represents the centre values of the nth hidden layer neurone, respectively.

### 5.2 Fuzzy stochastic improvement of the RBF neural network

The traditional RBF neural networks feature a simple network structure, straightforward output, and high computation speed. However, the model depends heavily on the parameters to be learned; therefore, the appropriateness of the parameter selection directly determines the network’s accuracy and convergence speed.

In an RBF neural network structure, radial basis functions are typically used as activation functions, with distance functions representing the basis functions of specific hidden layer nodes. Each hidden neurone has a network data centre, and the radial basis function is symmetric about this centre. Therefore, improvements to the traditional RBF neural network involve the fuzzy splitting of neurones and stochastic adjustment of neurone connections, optimising the algorithm to better handle uncertainties in engineering applications.

#### 5.2.1 Fuzzy splitting of neurones.

The activity level Af of hidden neurone *i*(*i = 1,2,…,k*) is computed using Equation (12).


$$Af_{i}=\frac{\text{1}}{\|x-{\tilde{\mu }}_{i}\|+\tau }\cdot \frac{\theta_{i}\left(x\right)}{\sum_{i=1}^{K}\theta_{i}\left(x\right)}\text{\textbackslash{}hspace\{0.17em}\;\;\;\;\;\;\;\;\}(i=1,2,\cdots ,K)$$ (12)

Where Af_i_ denotes the activation degree of the i-th hidden layer neurone, K denotes the number of neurones in the hidden layer, $\theta_{i}\left(x\right)$ refers to the output of the *i*-th neurone in the hidden layer, ${\tilde{\mu }}_{i}$ represents the fuzzy function for the data centre of the RBF network, and τ refers to a small real-valued number to prevent the activation function from becoming unsolvable when $\left|\right|x-{\tilde{\mu }}_{i}\left|\right|$ equals zero.

When the activation level Afi of neurone i exceeds the activation threshold Af0, the neurone i is considered active. The active neurone i is disconnected from the output neurone and then split, and the initial centre and variance of the new neurone are set accordingly.


$${\tilde{\mu }}_{ij}=\alpha_{j}{\tilde{\mu }}_{i}+\beta_{j}x$$ (13)

$${\tilde{\sigma }}_{ij}=\alpha_{j}{\tilde{\sigma }}_{i}\text{\textbackslash{}hspace\{0.17em}\;\;\;\;\;\;\;\}(j=1,2,\cdots ,N_{new})$$ (14)

Here, ${\tilde{\mu }}_{i}$ and ${\tilde{\sigma }}_{i}$ represent the fuzzy centre and fuzzy variance of neurone i, respectively. N_new_ is the number of newly added neurones determined by neurone activation levels. ${\tilde{\mu }}_{ij}$ and ${\tilde{\sigma }}_{ij}$ denote the fuzzy centre and fuzzy variance of neurone j, respectively. The value ranges of parameters $\alpha_{j}$ and $\beta_{j}$ are: $\alpha_{j}$ ∈ [0.95,1.05], $\beta_{j}$ ∈ [0,0.1]. The connection weight between the new neurone j and the output neurone is set as follows:


$${\tilde{\omega }}_{ij}=r_{j}\frac{{\tilde{\omega }}_{i}\cdot \theta_{i}\left(x\right)-e}{N_{\text{new}}\cdot \theta_{ij}\left(x\right)}$$ (15)

$$\sum_{j=1}^{N_{\text{new}}}{\text{r}}_{i}=1\text{\textbackslash{}hspace\{0.17em}\;\;\;\;\;\;\;\}(j=1,2,\cdots ,N_{new})$$ (16)

where r_j_ is the allocation parameter of the new neurone j, $\theta_{i}\left(x\right)$ is the output of the split neurone i, $\theta_{ij}\left(x\right)$ is the output of the newly split neurone j, ${\tilde{\omega }}_{i}$ denotes the fuzzy connection weight between the split neurone i and the output neurone, ${\tilde{\omega }}_{ij}$ is the fuzzy connection weight between the new neurone j and the output neurone, and e represents the current error in the neural network.

#### 5.2.2 Stochastic adjustment of neurone connection strength.

**Fig 17:**
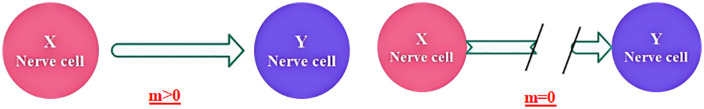
Adjustment of neurone connection modes.


The adjustment of neural connections mainly involves reselecting connections between existing neurones. According to Equations (17 ~ 18), the stochastic connection strength m of the information exchange between the hidden layer neurones and output layer neurones is calculated. In an RBF neural network, a large value of m indicates a strong information interaction between the hidden and output neurones, and such connections can be retained, as shown in Fig 17(a). When the value of m is small or approaches zero, this indicates a weak information interaction between the neurones. In such cases, the connections can be ignored during adjustment of the RBF network structure, as shown in Fig 17(b), thereby reducing the redundancy of the RBF neural network.

$$m(X;Y)=\frac{M(X;Y)}{min\left(H\left(X\right),H\left(Y\right)\right)}$$ (17)

$$M(X;Y)=\sum_{x,y}p\left(X,Y\right){log}_{\text{2}}\frac{p\left(X,Y\right)}{p\left(X\right)p\left(Y\right)}$$ (18)

In this context, p(X,Y) is the joint distribution density of neurones X and Y; p(X) and p(Y) are the probability densities of neurones X and Y, respectively; and H(X) and H(Y) denote the Shannon entropies of neurones X and Y, respectively.

If the stochastic connection strength m (j,y) of exchanged information between hidden layer neurone j and output layer neurone y is less than λ (where λ is the preset threshold for interaction strength), then the connection between neurone j and neurone y is disconnected. The hidden layer neurone closest to neurone j in terms of Euclidean distance, denoted as neurone j-j, is identified. The parameters of neurones j-j are as follows:


$$\mu {\text{'}}_{j-j}=\mu_{j-j}$$ (19)

$$\sigma {\text{'}}_{j-j}=\sigma_{j-j}$$ (20)

$$\omega {\text{'}}_{j-j}=\omega_{j}\frac{\theta_{j}\left(x\right)}{\theta_{j-j}\left(x\right)}$$ (21)

Where ω_j-j_, μ_j-j_, and σ_j-j_ denote the connection weight, centre, and variance of neurone j-j prior to structure adjustment, respectively, and ω´_j-j_, μ´_j-j_, and σ´_j-j_ denote the stochastic connection weight, centre, and variance of neurone j-j after structure adjustment, respectively. θ_j(x)_ and θ_j-j(x)_ correspond to the outputs of hidden layer neurones j and j-j before adjustment, respectively.

**Fig 18:**
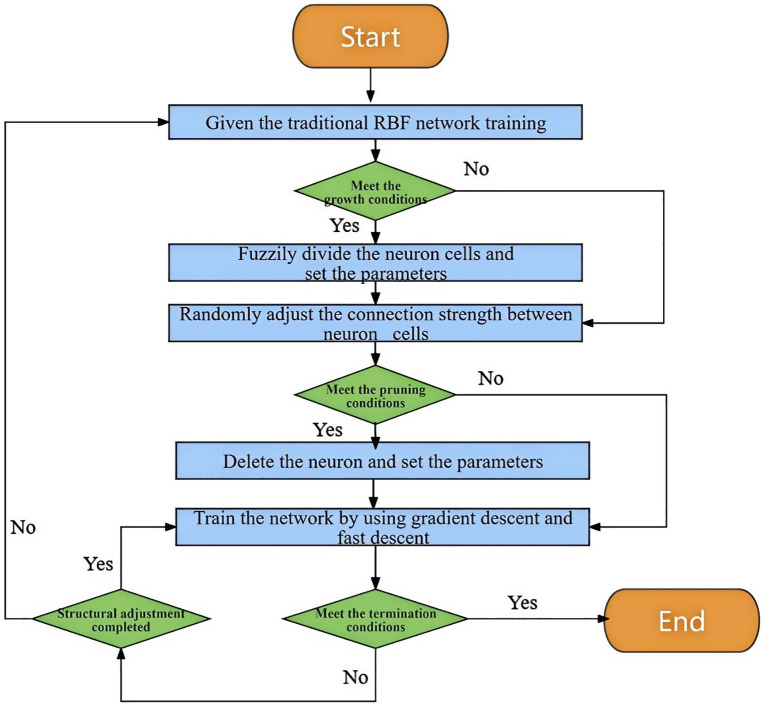
Flowchart of the improved RBF-based fuzzy stochastic prediction algorithm.

A flowchart of the improved RBF fuzzy neural network algorithm is shown in Fig 18.

## 6. Improved RBF fuzzy stochastic neural network abrasivity characteristics prediction model

### 6.1 Model development and determination of input and output variables

**Fig 19:**
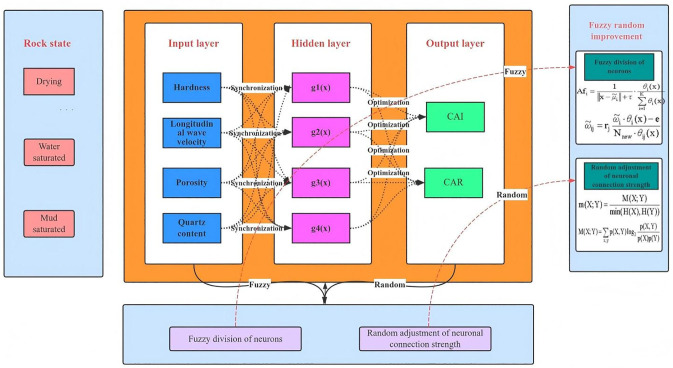
Prediction model of abrasiveness of WCS under different states.

According to the abrasivity test results, the TBM rock-breaking process in WCS is complex, and both the CAI and CAR vary with changing states, showing significant uncertainty. To achieve more accurate prediction of abrasive characteristics, an improved RBF fuzzy stochastic neural network model should be employed for training to capture the complex relationships between input and output sample vectors; the model topology is shown in Fig 19 [41–42].

The fuzzy stochastic RBF network integrates fuzzy c-means (FCM) clustering and probabilistic perturbation to model rock abrasivity’s fuzzy stochastic nature:

(1) Fuzzy neuron splitting: Based on FCM theory, hidden neurons are fuzzy clusters of input features (membership degree *μ*_*ij*_ (Equation(13)), with the fuzzy threshold (0.95–1.05) weighting parameter updates by membership (prioritizing neurons matching the sample’s feature distribution).
(2) Stochastic connection adjustment: A Gaussian perturbation weight model is adopted to simulate the spatial variation of rock wear rate (with a standard deviation of 0.025, consistent with the variation range of field observations). The perturbation is constrained through mutual information (estimated using kernel density estimation on a limited sample) to remain within the range relevant to engineering.
(3) Uncertainty reflection: Ambiguity can capture the saturation degree of the rock, while the stochastic model can simulate the stochastic fluctuations of the properties – when combined, they can yield a abrasivity rate distribution that matches the actual uncertainty during tunnel excavation.

Based on the collection, organisation, and analysis of experimental data, it was observed that the uncertainty of the abrasivity index of WCS in the western regions, whether in dry, water-saturated, or mud-saturated states, was influenced by factors such as hardness, wave velocity, porosity, and equivalent quartz content. Therefore, the input layer parameters of the model were set to these four variables.

Analysis of the abrasive characteristics of WCS in the western regions is essential for ensuring mechanical rock-breaking efficiency and tool wear control. The core issue is the accurate and effective determination of the CAI and the CAR of sandstones under different states. By developing an improved RBF fuzzy stochastic neural network model, the goal is to predict the abrasivity characteristics of sandstone in different states more accurately, providing a reference for TBM rock-breaking projects in terms of efficiency assessment, tool wear prediction, and tool selection. Accordingly, the output layer parameters of the prediction model were set as the CAI and CAR of WCS.

### 6.2 Determination of hidden layer units

In RBF neural networks, the selection of the number of hidden layer units is critical. If the number of hidden units is too small, the entire network cannot effectively train the samples or process the information. An excessive number of hidden units directly leads to structural redundancy and local minima. To balance these two aspects, the number of hidden units in a predictive network model is typically determined by using the following formula:


$$Z\text{=}\sqrt{mn+1.68n+0.93}$$ (22)

where Z is the number of hidden layer units, *n* is the number of network input variables, and *m* is the number of network output variables.

Based on the number of input and output variables in this prediction model, substituting into Equation (22) yields Z = 3.95; therefore, the number of hidden layer units in the fuzzy stochastic RBF network model was set to four.

### 6.3 Training and learning of the improved rbf network prediction model

Considering the geological and geomorphological similarities in the western region, to avoid deviations in the network training results caused by input sample differences, all training sample data were selected from WCS in the same stratum based on physical and mechanical parameters measured in dry, water-saturated, and mud-saturated states. The hardness, wave velocity, porosity, and equivalent quartz content were used as input variables, as listed in Table 3. According to the principles of the improved RBF fuzzy stochastic network algorithm, the measured abrasivity index CAI and abrasivity ratio CAR of the sample set were fully utilised for adjustment and training, with the learning parameter settings listed in Table 4. The predicted data was considered valid when the training sample deviation was within 5%.

**Table 3:** Predictive model training data.

Number	Hardness (HL)	Wave velocity (km·s^-1^)	Porosity (%)	Equivalent quartz content (%)	CAI	CAR
C1-1	361	2.247	6.68	63.06	0.43	0.613
C1-2	366	2.069	6.71	63.81	0.46	0.584
C1-3	369	2.222	6.68	64.00	0.48	0.446
C1-4	372	2.282	7.07	63.67	0.49	1.612
C1-5	373	2.353	6.15	64.35	0.62	0.399
C1-6	381	2.532	6.40	64.20	0.64	0.201
C2-1	323	2.724	7.04	70.73	0.28	2.592
C2-2	319	2.868	6.95	71.09	0.36	3.154
C2-3	320	3.02	6.85	71.13	0.38	2.639
C2-4	329	2.835	6.97	71.10	0.42	0.873
C2-5	330	2.786	6.83	71.11	0.50	3.011
C2-6	339	3.162	6.53	71.69	0.55	4.468
C3-1	331	2.728	7.18	70.49	0.33	2.338
C3-2	337	2.874	7.06	70.67	0.40	1.634
C3-3	329	3.098	7.31	70.54	0.42	0.872
C3-4	346	2.798	6.89	71.57	0.45	1.203
C3-5	342	2.822	6.70	72.87	0.59	0.990
C3-6	354	3.210	6.84	72.13	0.63	0.497

**Table 4:** Network training parameter settings.

Parameter	Learning rate	Forgetting factor	Training error	Number of hidden elements	Interaction information strength threshold
Number	0.25	0.15	5%	4	0.90

After completing the preparatory work, the sample data were used for training. Table 5 lists the training results of the abrasivity index prediction model for sandstone in different states. After training with the CAI sample data of the WCS from the western region, the predicted CAI and CAR values from the improved RBF fuzzy neural network model closely matched the measured values, with maximum deviations of 4.19% and −4.24%, respectively. The relative errors were all well controlled to within 5%. Therefore, the improved network prediction model effectively captures the complex relationships between the input and output and can serve as a reliable predictive tool for abrasivity characteristics in TBM rock-cutting engineering.

**Table 5:** Training results for abrasive properties.

States	Number	Output value and error			
		CAI output value	CAI error (%)	CAR output value	CAR error (%)
Dry	C1-1	0.446	3.79	0.597	−2.68
	C1-2	0.449	−2.45	0.602	3.15
	C1-3	0.500	4.12	0.437	−1.98
	C1-4	0.508	3.66	1.546	−4.07
	C1-5	0.632	2.01	0.408	2.25
	C1-6	0.619	−3.21	0.207	3.03
Water- saturated	C2-1	0.292	4.19	2.482	−4.24
	C2-2	0.373	3.51	3.060	−2.99
	C2-3	0.367	−3.32	2.688	1.86
	C2-4	0.437	4.04	0.902	3.37
	C2-5	0.516	3.23	2.917	−3.11
	C2-6	0.535	−2.76	4.559	2.04
Mud- saturated	C3-1	0.340	2.98	2.299	−1.65
	C3-2	0.413	3.32	1.695	3.74
	C3-3	0.403	−4.15	0.844	−3.20
	C3-4	0.468	3.96	1.228	2.09
	C3-5	0.579	−1.79	0.963	−2.68
	C3-6	0.647	2.62	0.509	2.33

### 6.4 Engineering verification of the improved RBF network prediction model

**Fig 20:**
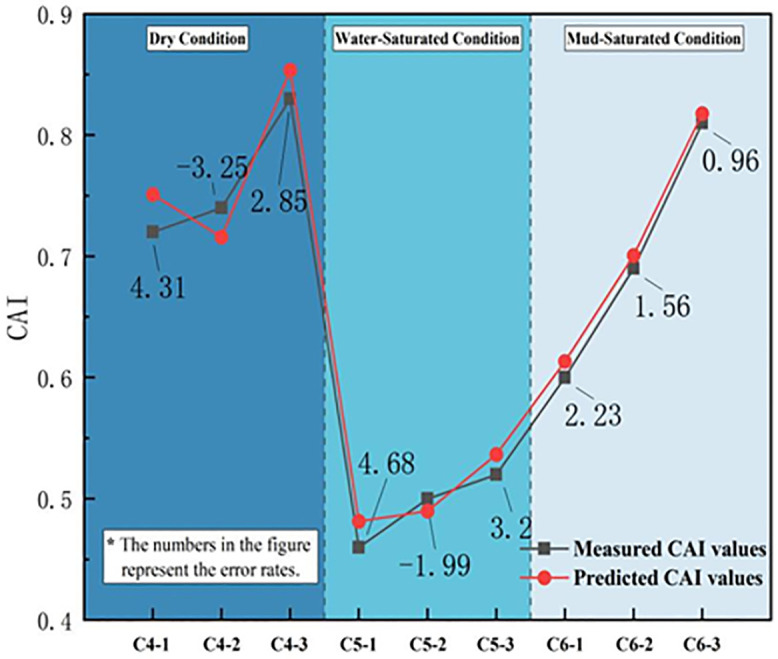
Comparison between the CAI measured value and the model predictions.

**Fig 21:**
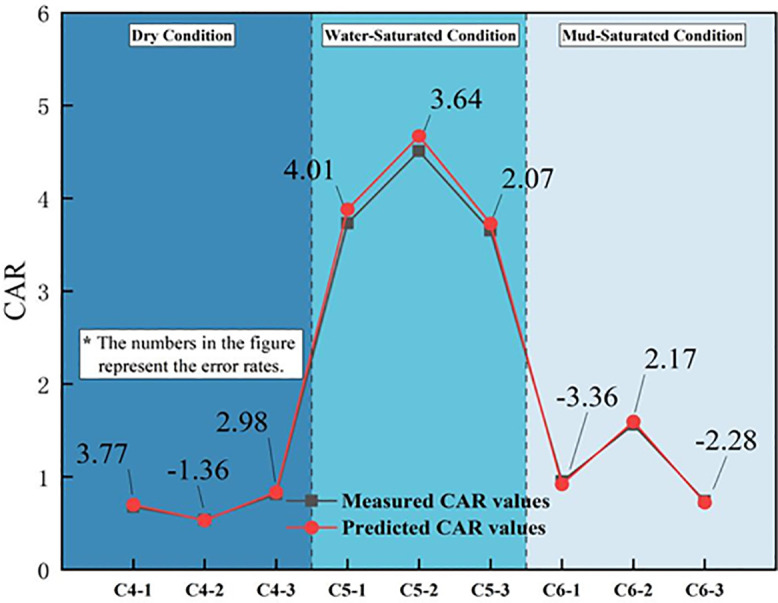
Comparison between the CAR measured value and the model predictions.

To further verify the reliability of the improved RBF network prediction model for forecasting abrasivity characteristics, control group tests were conducted using WCS from similar strata in comparable TBM projects. Three sets of tests were performed for each state in a total of nine groups. The training data samples for the verification groups are shown in Table 6. The CAI and CAR values predicted by the network model were compared with the actual values using a relative error of 5% as the acceptance criterion. The corresponding results are listed in Table 7. Additionally, comparison plots between the predicted and actual CAI values and between the predicted and actual CAR values are shown in Figs 20 and 21, respectively.

**Table 6:** Validation group training data samples.

Number	Hardness (HL)	Wave velocity (km·s^-1^)	Porosity (%)	Equivalent quartz content (%)
C4-1	422	2.762	7.17	73.67
C4-2	421	2.843	6.24	74.35
C4-3	433	3.042	6.49	74.2
C5-1	378	3.335	7.07	81.1
C5-2	381	3.286	6.93	81.11
C5-3	389	3.662	6.63	81.69
C6-1	395	3.298	6.99	81.57
C6-2	393	2.822	6.8	82.87
C6-3	404	3.71	6.94	82.13

**Table 7:** Prediction results for abrasive properties.

States	Number	Output value and error					
		CAI measured values	CAI predictions	CAI error (%)	CAR measured values	CAR predictions	CAR error (%)
Dry	C4-1	0.72	0.701	−2.62	0.676	0.701	3.77
	C4-2	0.74	0.769	3.98	0.539	0.532	−1.36
	C4-3	0.83	0.841	1.32	0.812	0.836	2.98
Water- saturated	C5-1	0.46	0.472	2.55	3.732	3.882	4.01
	C5-2	0.50	0.459	−4.37	4.507	4.671	3.64
	C5-3	0.52	0.609	3.20	3.652	3.728	2.07
Mud- saturated	C6-1	0.60	0.726	2.27	0.953	0.921	−3.36
	C6-2	0.69	0.729	−2.76	1.562	1.596	2.17
	C6-3	0.81	0.787	3.53	0.741	0.724	−2.28

According to the prediction results of the validation group for sandstone abrasivity characteristics, the predicted values from the improved RBF fuzzy stochastic neural network closely match the measured values, with a maximum error of −4.37%, all controlled within 5%. This indicates that the prediction model achieves reliable engineering forecasting of the uncertainty distribution of the abrasivity characteristics of WCS in the western region and provides valuable references for TBM mechanical rock-breaking efficiency evaluation, tool wear prediction, and tool selection.

To verify the performance of the improved algorithm, engineering predictions were conducted using the improved RBF fuzzy neural network algorithm, the traditional RBF, the multiple linear regression, and support vector regression model respectively. Their efficiencies were compared over 1000 iterations. The test results is shown in Table 8. The specific configuration of the experimental platform host was as follows: CPU Intel Core i5-6500, 3.2 GHz clock speed, 256GB DDR memory, 1TB hard drive, and 1000M network card. The software platform used was Windows 10 SP3, and the debugging software was MATLAB 2010B. As shown in Table 8, the improved fuzzy stochastic RBF outperforms baseline models: its MSE/MAE are reduced by 62.0%/47.4% (compared to MLR) and 65.7%/47.0% (compared to SVR), while the R is increased by 15.4%/13.5%. Compared with the traditional RBF model, the improved RBF also shows significant advantages: its MSE is reduced by 60.6%, MAE is reduced by 44.4%, and R²is increased by 12.0%. Meanwhile, the occurrence rate of local optimum is reduced by 53.8%. This confirms that the proposed model has stronger non-linear fitting ability and robustness than both simple linear models, mainstream non-linear baseline methods, and the classical RBF network.

**Table 8:** Comparison of performance indicators of different models.

Algorithm type	Total number of runs	Occurrence rate of local optimum (%)	Mean squared error (MSE)	Mean absolute error (MAE)	Coefficient of determination (R^2^)	10-fold cross- validation MSE	10-fold cross- validation R²
Multiple Linear Regression (MLR)	1000	45.71	8.62	2.58	0.821	9.01	0.812
Support Vector Regression (SVR)	1000	35.17	5.92	2.13	0.852	6.25	0.843
Traditional RBF	1000	28.69	5.15	1.89	0.863	5.47	0.851
Improved RBF	1000	13.25	2.03	1.05	0.967	2.18	0.959

To address overfitting risks from the small sample size, 10-fold cross-validation was performed. The improved RBF model achieved an average cross-validation R^2^ of 0.959 (Table 8), indicating stable performance across different data partitions.

## 7. Discussion

This study investigates the abrasivity laws of WCS in western China under dry, water-saturated, and mud-saturated states, and establishes an improved RBF fuzzy stochastic neural network model for prediction. Here, we discuss the main findings, their engineering implications, and research limitations.

(1) The CAI of WCS is the highest in the dry state, followed by the mud-saturated state, and the lowest in the water-saturated state, with quantitative differences. This phenomenon is closely related to the microstructural changes of WCS under different water-bearing states: in the dry state, the internal structure of WCS is intact, the cementation degree is high, and the rock strength is strong, so the abrasivity effect on the Cerchar steel needle is more significant. In the water-saturated state, water infiltrates into the rock pores, weakening the cementation between particles, reducing the rock’s hardness and abrasiveness, thus lowering the CAI.
(2) Regarding the CAR, the order is water-saturated state > mud-saturated state > dry state, which is consistent with the microstructural analysis results. The intact structure and high strength of dry WCS make it difficult for the steel needle to abrade, resulting in low CAR. While the water-saturated state reduces the wear resistance of the rock, it leads to a higher wear efficiency of the steel needle.
(3) The improved RBF fuzzy stochastic neural network model, optimized by neuron fuzzy splitting and stochastic adjustment of connection strength, effectively addresses the limitations of traditional RBF networks in handling engineering uncertainty. By using rock hardness, wave velocity, porosity and equivalent quartz content as input parameters, this model can make highly accurate predictions for CAI and CAR, effectively enhancing the ability to capture the nonlinear relationship between rock physical properties and wear characteristics.
(4) The research results have important guiding significance for TBM construction in western China, where WCS is widely distributed. The quantitative relationship between water-bearing states and abrasivity characteristics can help engineers adjust construction strategies to reduce tool wear. The high-precision prediction model can quickly predict the abrasivity characteristics of WCS without tedious tests, providing a basis for TBM tool selection, wear prediction, and construction efficiency evaluation. This is particularly important for improving the safety and economy of TBM construction in weak rock strata.
(5) The sample size in this study was limited. Future research should increase the sample size and cover WCS from different regions to enhance the universality of the results. Additionally, the small sample size (constrained by experimental costs) limits the model’s generalizability to weakly cemented sandstones with extreme mineral compositions or stress states. Future work will expand the dataset to broaden the conclusion’s applicability.

## 8. Conclusion

(1) Cerchar abrasivity characteristic tests on WCS from the western region revealed that the abrasivity properties of sandstone varied with its state and exhibited significant engineering uncertainty. Overall, the abrasivity index was the highest in the dry state, followed by the mud-saturated state, and was the lowest in the water-saturated state. The abrasivity index in the dry state was 10.64% higher than that in the mud-saturated state, and 19.23% higher than that in the water-saturated state.
(2) Studies on the micro-characteristics of WCS under different states revealed that the abrasivity efficiency evaluation index, CAR, follows the order: water-saturated mud-saturated state, and dry state. Because of the sandstone’s intact internal structure, high cementation, and high strength, the abrasivity index CAI of WCS in the dry state was higher than those in the water-saturated and mud-saturated states. During the mud-saturation process, the steel needle experienced greater wear during abrasivity than in the water-saturated state because a layer of mud enveloped the sandstone surface.
(3) To address the shortcomings of the traditional RBF neural network algorithm in handling engineering uncertainty problems, neurone fuzzy splitting and stochastic adjustment of the neurone connection strength were adopted to optimise the network model. Using rock hardness, wave velocity, porosity, and equivalent quartz content as inputs, and the abrasivity index CAI and abrasivity ratio CAR of WCS as outputs, with four hidden layer units, an improved RBF fuzzy stochastic neural network abrasivity characteristic prediction model was established. The improved fuzzy stochastic RBF model has achieved the following improvements compared to MLR, SVR, and traditional RBF: the MSE is reduced by up to 65.7%, the MAE is reduced by up to 47.4%, and the occurrence rate of local optimality is decreased by 53.8%.
(4) Engineering cases demonstrate that the improved RBF fuzzy stochastic neural network prediction model, after effective training, can produce predicted values of the CAI and CAR that closely match the measured engineering values, with errors within 5%. This indicates that the prediction model achieves good engineering forecasting of the uncertainty distribution of the abrasivity characteristics of WCS in the western region, providing an important reference value for the evaluation of TBM mechanical rock-breaking efficiency and tool wear prediction.
